# Assessing Brain Lesions and Predicting Functional Outcome in Patients With Anterior Circulation Stroke With Endovascular Therapy: Co‐ASPECTS

**DOI:** 10.1161/JAHA.126.051171

**Published:** 2026-08-20

**Authors:** Jiaqi Luo, Xiaolin Zhao, Mengxuan Xiao, Guoshuai Yang, Pengxiang Li, Weihua Liu, Suyue Pan, Yidong Deng, Xianghong Liu, Kaibin Huang

**Affiliations:** ^1^ Department of Neurology Ganzhou Hospital‐Nanfang Hospital, Southern Medical University Ganzhou Jiangxi Province China; ^2^ Department of Neurology Nanfang Hospital, Southern Medical University Guangzhou Guangdong Province China; ^3^ Department of Neurology Central South University Xiangya School of Medicine Affiliated Haikou Hospital Haikou Hainan Province China; ^4^ Department of Neurology The Second Affiliated Hospital of Hainan Medical University Haikou Hainan Province China; ^5^ Department of Encephalopathy Guangdong Provincial Hospital of Chinese Medicine, Hainan Hospital Haikou Hainan Province China; ^6^ Department of Neurology Hainan General Hospital, Hainan Affiliated Hospital of Hainan Medical University Haikou Hainan Province China

**Keywords:** ASPECTS, endovascular therapy, functional outcome, ischemic stroke, NCCT, Ischemic Stroke, Cerebrovascular Procedures, Computerized Tomography (CT), Prognosis

## Abstract

**Background:**

This study aimed to develop and validate a practical and reliable tool for comprehensive assessment of brain lesions on immediate post‐endovascular therapy (EVT) noncontrast computed tomography (CT) and to predict 90‐day functional outcomes in patients with acute ischemic stroke.

**Methods:**

We retrospectively reviewed consecutive patients with acute ischemic stroke undergoing EVT from 2 academic hospitals as the development cohort and used an independent national multicenter prospective cohort for external validation. The new score was derived from the Alberta Stroke Program Early CT Score (ASPECTS) on the immediate post‐EVT non‐contrast CT, integrating both hyperdensity and hypodensity signs, named comprehensive Alberta Stroke Program Early CT Score (Co‐ASPECTS). Unfavorable outcome was defined as modified Rankin Scale score of 3 to 6 at 90 days.

**Results:**

A total of 1015 patients were included (development cohort: 542; validation cohort: 473). Co‐ASPECTS, formulated through equal‐weight summation of hyperdensity and hypodensity signs and consistent with the 0 to 10 score ASPECTS framework, was significantly associated with unfavorable outcomes (adjusted odds ratio, 0.45 [95% CI, 0.40–0.51]). It showed discrimination for functional outcome with a C‐statistic of 0.889 in the development cohort, outperforming conventional models including baseline ASPECTS and immediate post‐EVT ASPECTS that calculated based on hyperdensity or hypodensity signs alone. In the validation cohort, Co‐ASPECTS maintained robust performance (C‐statistic, 0.847). It also appeared to reflect both infarct burden and secondary injury, showing associations with final infarction and intracranial hemorrhage.

**Conclusions:**

Co‐ASPECTS may serve as a promising tool with reliability and practicality for assessing brain lesions and predicting functional outcomes of patients with acute ischemic stroke who underwent EVT in clinical practice.

**Nonstandard Abbreviations and Acronyms**
AIS: acute ischemic stroke
ASPECTS: Alberta Stroke Program Early Computed Tomography Score
BBB: blood–brain barrier
Co‐ASPECTS: Comprehensive Alberta Stroke Program Early Computed Tomography Score
mRS: modified Rankin Scale
NCCT: noncontrast computed tomography
NIHSS: National Institutes of Health Stroke Scale

**Clinical Perspective**

**What Is New?**

- The Comprehensive Alberta Stroke Program Early Computed Tomography Score is a newly derived scoring tool using immediate post‐endovascular therapy noncontrast computed tomography that integrates both hyperdensity and hypodensity signs, providing a more accurate prediction of 90‐day functional outcomes in patients with anterior circulation stroke than conventional models using either indicators or baseline imaging, which was confirmed by both internal and external validation.

**What Are the Clinical Implications?**

- By comprehensively reflecting both primary ischemic burden and secondary procedural injury, Comprehensive Alberta Stroke Program Early Computed Tomography Score provides clinicians with an early, reliable, highly accessible bedside tool for brain lesion assessment and outcome prediction following endovascular therapy.
- Comprehensive Alberta Stroke Program Early Computed Tomography Score serves as a reference for early risk stratification using routine post‐endovascular therapy imaging without additional examination, helping to identify patients with low scores who suffer more severe injuries and are unlikely to achieve functional independence needing closer monitoring and more aggressive treatment, whereas high scores predict favorable outcomes that may guide less intensive monitoring and earlier rehabilitation.

Endovascular therapy (EVT) has been proven to be a revolutionary treatment for acute ischemic stroke (AIS).^1^, ^2^ The achievement of successful recanalization through EVT is associated with a substantially increased probability of getting a favorable outcome, even among those with serious conditions.^3^, ^4^, ^5^ However, a significant proportion of patients remain disabled or die after EVT, making the prediction of clinical outcomes a critical issue.^5^, ^6^, ^7^ The prognosis for patients with AIS undergoing EVT is multifactorial, influenced not only by the initial severity of the stroke but also critically by the efficacy of the recanalization process. As a result, the condition that precedes recanalization may not reflect the brain lesions or predict the functional outcome accurately enough. A more optimal time for prognostic assessment appears to be immediately after EVT. At this valuable time point, the influence of recanalization treatment could be fully assessed, providing a more accurate and still timely evaluation for patients' outcomes.

The Alberta Stroke Program Early Computed Tomography (CT) Score (ASPECTS) is a 10‐region scoring system pivotal for reflecting brain ischemic lesions in patients with AIS that evaluates the infarct extent through the hypodensity signs by noncontrast CT (NCCT).^8^ In addition to the primary ischemic injury, during EVT, the compromised integrity of ischemic brain tissue may be further damaged by the invasive interventional operation, pressure from contrast injection, or ischemic reperfusion injury per se, potentially leading to the blood–brain barrier (BBB) disruption and subsequent contrast extravasation or hemorrhagic transformation.^9^, ^10^, ^11^ Such pathologic damage manifests as hyperdensity signs on post‐EVT NCCT scans, with a clinical incidence ranging from 40% to 74%.^11^, ^12^, ^13^, ^14^, ^15^, ^16^ Moreover, delayed recanalization, lack of collateral circulation, and reperfusion injury may lead to exacerbated brain ischemia and cerebral edema presenting as an expansion of hypodensity range on post‐EVT NCCT. Therefore, post‐EVT NCCT not only reflects the cumulative cerebral insult after recanalization treatment but also serves as a visual reflection of the comprehensive brain's pathophysiological state following AIS onset. Previous study has applied ASPECTS to hyperdense lesion on NCCT immediately after EVT, showing a significant connection with functional outcome.^17^ We hypothesized that integrating the hyperdensity and hypodensity signs of post‐EVT NCCT could better reflect brain tissue damage and thus more accurately predict patient outcomes.

In this study, our objective was to develop a reliable and practical tool for the early assessment of comprehensive brain lesions in patients with AIS who have received EVT. This tool was based on the integration of immediate post‐EVT NCCT findings with the ASPECTS framework. We further evaluated its generalizability through external validation in an independent cohort.

## METHODS

The data sets used and analyzed during this study are available from the corresponding authors on reasonable request.

### Study Design and Population

The study population was divided into the development cohort and the validation cohort. In the development cohort, we retrospectively reviewed patients with AIS who received EVT and consecutively registered at 2 academic hospitals between January 2021 and April 2023. In the validation cohort, we included patients from a national, multicenter, prospective, observational cohort conducted at 19 hospitals in China, which aimed to observe the effect of prophylactic medication for stress ulcers in patients with AIS with acute anterior circulation large vessel occlusion undergoing EVT and launched in May 2023 (clinicaltrials.gov; NCT05845372). Compared with the development cohort, the validation cohort included only patients with baseline National Institutes of Health Stroke Scale (NIHSS) score ≥6, and there were no other differences between the 2 cohorts.

In this study patients were included if they (1) were aged ≥18 years; (2) received EVT for large vessel occlusion in the anterior circulation; (3) did not have significant disability before onset (premodified Rankin Scale [mRS] score ≤1); and (4) underwent NCCT examination immediately post‐EVT (within 1 hour). Patients were excluded if they (1) had poor‐quality CT images affecting judgment; (2) failed to follow up at 90 days after onset; and (3) had other serious illnesses resulting in a life expectancy of <3 months. Across all centers, selection of patients with AIS for EVT strictly followed contemporary American Heart Association/American Stroke Association guidelines.^18^ In both the development and validation cohorts, except for the preceding criteria, patients with anterior circulation large vessel occlusion AIS undergoing EVT were included if they had baseline NIHSS score ≥6, ASPECTS ≥6, and met 1 of the following time criteria: within (1) 6 hours of onset; (2) 6 to 16 hours with DEFUSE‐3 (Endovascular Therapy Following Imaging Evaluation for Ischemic Stroke 3) or DAWN (Clinical Mismatch in the Triage of Wake Up and Late Presenting Strokes Undergoing Neurointervention With Trevo) criteria; (3) 16 to 24 hours with DAWN criteria. In the retrospective development cohort drawn from large academic hospitals, a part of patients with baseline NIHSS score <6 who underwent EVT after individualized benefit–risk assessment were also included. The study protocol was approved by the Ethics Committee of Nanfang Hospital Southern Medical University (NFEC‐2023‐040). In both the development and validation cohorts, all participants had signed written informed consent to use their deidentified clinical data and imaging before their enrollment.

### Clinical and Imaging Data Collection

Patients' clinical records and imaging data were retrieved from the hospitals' electronic medical record system or electronic data capture system. The image evaluation was performed on the picture archiving and communication system. The unfavorable functional outcome was defined as a score of 3 to 6 on the mRS at 90 days. The mRS score was obtained through structured telephone interviews conducted by 2 neurologists blinded to all imaging and clinical data.

In this study, the ASPECTS scoring system was employed to quantitatively evaluate hyperdensity and hypodensity signs on NCCT scans obtained immediately post EVT. Briefly, the ASPECTS scoring system is divided into the following 10 regions: caudate, internal capsule, lentiform, insular ribbon, anterior middle cerebral artery cortex, middle cerebral artery cortex lateral to the insular ribbon, posterior middle cerebral artery cortex, and the anterior, lateral, and posterior middle cerebral artery territories immediately superior to rostral to basal ganglia.^8^ For post‐EVT NCCT, each region with hyperdensity or hypodensity receives 1 point; the total score (0–10) ranges from mildest (0) to most severe (10). The same scoring applies to both signs (Figure 1). The CT scans were independently evaluated by 2 senior neuroradiologists blinded to all clinical data, with any regional discrepancies resolved through consensus by a third blinded senior expert. Interobserver reliability was quantified using the Cohen's kappa coefficient and intraclass correlation coefficient. The region‐by‐region Cohen's kappa coefficient was 0.890, and the intraclass correlation coefficient for the total Comprehensive ASPECTS (Co‐ASPECTS) was 0.940 (95% CI, 0.912–0.959). Cohen's kappa of hypodensity signs and hyperdensity signs was 0.849 and 0.924. The potential direct summation scores and coefficient‐based weighted scores were used in sensitivity analyses to compare the performance of the constructed scoring systems.

**Figure 1:**
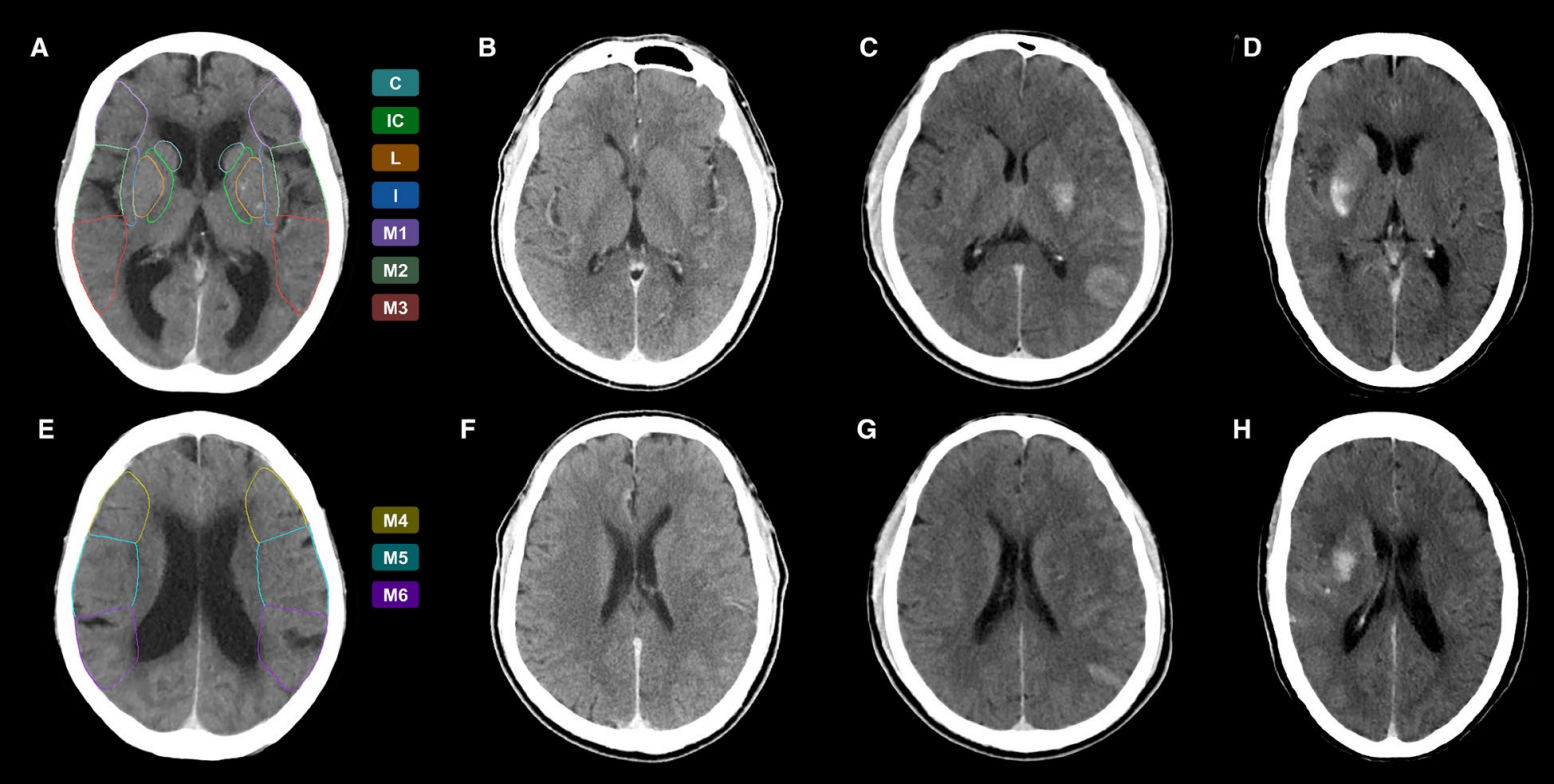
Representative cases of ASPECTS scoring system in this study. **A** and **E**, The 10 regions according to the ASPECTS scoring system. **B** and **F**, The NCCT images of a patient immediately post EVT with hypodensity signs in the M2 and M5 regions. **C** and **G**, The NCCT images of a patient immediately post EVT with hyperdensity signs in the M2, M3, L, and M6 regions. **D** and **H**, The NCCT images of a patient immediately post EVT with hypodensity signs in the I region and hyperdensity signs in the L and M5 regions, with the final Co‐ASPECTS of 7. C indicates caudate; Co‐ASPECTS, Comprehensive Alberta Stroke Program Early Computed Tomography Score; EVT, endovascular thrombectomy; I, nsular ribbon; IC, internal capsule; L, lentiform; MCA, middle cerebral artery; M1, anterior MCA cortex; M2, MCA cortex lateral to insular ribbon; M3, posterior MCA cortex; M4, M5, and M6, anterior, lateral, and posterior MCA territories immediately superior to M1, M2, and M3, rostral to basal ganglia; and NCCT, noncontrast computed tomography.

Head‐to‐head comparison would be performed using 2 well‐validated AIS scores: the Pittsburgh Response to Endovascular Therapy (PRE) score and the Totaled Health Risks in Vascular Events (THRIVE) score.^19^, ^20^ PRE score was calculated as age (years)+2×baseline NIHSS score − 10×baseline ASPECTS. The THRIVE score was computed by summing categorical points assigned to age (0, <60 years; 1, 60–79 years; 2, ≥80 years), baseline NIHSS score (0, 0–10; 2, 11–20; 4, ≥21), and 3 comorbidities (hypertension, diabetes, atrial fibrillation 1 point each).

In most patients for whom only NCCT was available, final infarct size was measured using the previously reported method by NCCT ∼24 hours after EVT.^21^, ^22^ To maintain comparability across different time points within this study, the final infarction was quantified using the ASPECTS scoring system 24±6 hours after EVT.

### Statistical Analysis

In this study, variables that were nonnormally distributed and continuous were expressed as the median and interquartile range, and categorical variables were presented as counts and percentages. Variables with *P* value <0.1 in univariate analysis were identified as potential predictors and then were analyzed using multivariable logistic regression. This approach could avoid omitting critical confounders that may become significant or alter effect estimates only after multivariable adjustment. A basic predictive model using statistically significant predictors was constructed to serve as a reference for comparison of our scoring model.

Before finalizing the scoring system we assessed the hyperdensity–hypodensity interaction on additive and multiplicative scales to test whether their coexistence adds prognostic value beyond individual effects and supports their integration into a single score. Receiver operating characteristic curve analysis was used to evaluate the predictive accuracy and the Hosmer–Lemeshow test and Brier score were used to examine the fit of the model. Decision curve analysis was used as a complementary assessment to quantify the net benefit of models across threshold probabilities for outcome, with higher net benefit indicating greater clinical utility. The net reclassification index and integrated discrimination improvement indices were used to quantify the improvement in predictive performance after the addition of our scoring model. Subgroup analyses were performed at different ages, sex, hemorrhagic transformation, occlusion vessels, recanalization, and stroke severity, where age was dichotomized at 65 years, a standard cutoff for high cardiovascular risk, and baseline NIHSS scores stratified stroke severity into mild (<5), moderate (5–15), and severe (>15) groups.^23^, ^24^

Multiple imputation by chained equations was implemented using the mice package in R, generating 20 imputed data sets (m=20) after 50 iterations (maxit=50). The imputation model included all analysis variables, with predictive mean matching applied for imputation. Results were pooled across imputed data sets using Rubin's rules.^25^

All statistical analyses were conducted using SPSS version 25 (IBM, Armonk, NY, USA) and R 4.2.2. The statistical significance level for all tests was set at *P*<0.05.

## RESULTS

### Study Population

We finally included a total of 1015 patients with AIS who underwent EVT, including 542 in the development cohort and 473 in the validation cohort (Figure S1). Of the patients in the development cohort, 171 (32%) were women, the median baseline NIHSS score was 10 (interquartile range, 7–15), and 271 (50%) had unfavorable functional outcomes (mRS score 3–6) (Table 1). Of the patients in the validation cohort, 148 (31%) were women, the median baseline NIHSS score was 13 (interquartile range, 10–17), and 267 (56%) had unfavorable functional outcomes (Table S1).

**Table 1:** Patient Characteristics of the Development Cohort

Characteristics	Overall population (n=542)	90‐d mRS score ≤2 (n=271)	90‐d mRS score ≥3 (n=271)	SMD	*P* value
Age, y, median (IQR)	65 (56–73)	65 (56–72)	65 (56–73)	0.044	0.653
Male sex, n (%)	371 (68)	173 (64)	198 (73)	0.199	0.021*
Underlying diseases, n (%)					
Hypertension	321 (59)	155 (57)	166 (61)	0.083	0.336
Diabetes	145 (27)	78 (29)	67 (25)	−0.092	0.286
Dyslipidemia	155 (29)	61 (23)	94 (35)	0.272	0.002*
Prior stroke	84 (16)	47 (17)	37 (14)	−0.102	0.235
Atrial fibrillation	98 (18)	47 (17)	51 (19)	0.038	0.655
Prestroke mRS score 0, n (%)	454 (84)	216 (80)	238 (88)	0.221	0.010*
Received i.v. tPA, n (%)	123 (23)	60 (22)	63 (23)	0.026	0.758
Clinical parameters at admission					
Systolic BP, mm Hg, median (IQR)	143 (129–158)	141 (129–156)	145 (130–161)	0.129	0.067
Diastolic BP, mm Hg, median (IQR)	82 (75–92)	82 (76–90)	83 (74–93)	0.027	0.798
Glucose, mmol/L, median (IQR)	7.0 (5.7–8.7)	7.1 (5.7–8.9)	6.9 (5.8–8.5)	−0.118	0.620
National Institutes of Health Stroke Scale score, median (IQR)	10 (7–15)	8 (4–13)	13 (9–17)	0.739	<0.001*
Alberta Stroke Program Early Computed Tomography Score, median (IQR)	8 (7–9)	9 (8–9)	8 (6–9)	−0.603	<0.001*
Occlusion location, n (%)					
ICA	148 (27)	82 (30)	66 (24)	−0.133	0.123
MCA	323 (60)	164 (61)	159 (59)	−0.038	0.836
ICA and MCA	71 (13)	25 (9)	46 (17)	0.231	0.008*
Onset‐to‐recanalization, min, median (IQR)	545 (368–917)	495 (340–861)	624 (430–950)	0.228	<0.001*
Stroke subtype per Trial of Org 10 172 in Acute Stroke Treatment, n (%)					
Large artery atherosclerosis	336 (62)	178 (66)	158 (58)	−0.152	0.077
Cardioembolism	134 (25)	58 (21)	76 (28)	0.154	0.073
Other	72 (13)	35 (13)	37 (14)	0.022	0.800
Modified Thrombolysis in Cerebral Infarction post endovascular therapy, n (%)					
0‐2a	40 (8)	12 (4)	28 (10)	0.227	0.009*
2b‐2c	143 (26)	61 (23)	82 (30)	0.177	0.041*
3	359 (66)	198 (73)	161 (60)	−0.292	<0.001*

BP indicates blood pressure; ICA, internal cerebral artery; IQR, interquartile range; MCA, middle cerebral artery; mRS, modified Rankin Scale; and SMD, standardized mean difference.

* Statistically significant.

### Construction of Comprehensive ASPECTS

We categorized patients into 2 groups based on functional outcomes. On performing univariate analyses in the development cohort, we found that patients who had unfavorable outcomes tended to be more male, had more dyslipidemia, poorer preonset condition, longer time to recanalization, poorer degree of recanalization, higher baseline NIHSS scores, and lower baseline ASPECTS (Table 1). In addition, we compared the hyperdensity and hypodensity sign scores following the ASPECTS scoring system in the immediate post‐EVT NCCT scans. Notably, patients with unfavorable outcome exhibited significantly elevated scores for both hyperdensity (odds ratio [OR], 2.36 [95% CI, 2.00–2.79]; *P*<0.001) and hypodensity signs (OR, 2.12 [95% CI, 1.82–2.46]; *P*<0.001), and the association was retained after adjustment for potential confounders (Table S2).

Furthermore, to elucidate the individual impacts of contrast extravasation and intracranial hemorrhage, a subgroup analysis was conducted among patients presenting with hyperdensity. Multivariable logistic regression revealed that both groups were statistically significantly associated with unfavorable outcomes (adjusted OR, 1.95 [95% CI, 1.47–2.59] and adjusted OR, 1.88 [95% CI, 1.32–2.66]) (Table S3). In another subgroup analysis, patients with contrast extravasation showed no significant difference in final infarction versus those without hyperdensity signs but had a significantly higher risk of unfavorable outcome (OR, 2.54 [95% CI, 1.88–3.43]) (Table S4).

The Co‐ASPECTS was developed based on the scores of hyperdensity and hypodensity signs. Initially, we assessed the interaction between these 2 scores, confirming a significant additive interaction effect that suggested a combined influence on functional outcome with greater association (Table S5). Subsequently, the strength of their association with the outcome across different models controlling for multiple confounders was evaluated. The analysis revealed a consistently approximate 1‐to‐1 effect ratio between the scores of hyperdensity and hypodensity signs (Table S2). Based on these results, we adopted an equal weighting approach, summing the scores of the hyperdensity and the hypodensity sign to formulate the Co‐ASPECTS. To align with the conventional ASPECTS methodology, we stipulated that the identification of hyperdensity or hypodensity signs within any of the designated 10 cerebral regions would result in a 1‐point decrement from the full score of 10. Hence, a score of 10 denoted the mildest severity, and a score of 0 indicated the highest severity level. Specially, when both hyperdensity and hypodensity signs were present in a region, only one point was deducted to avoid double counting and maintain consistency with the original 0 to 10 ASPECTS scale.

In all ASPECTS‐regions of total population, the regional co‐occurrence of hyperdensity and hypodensity signs frequency was 0.66% (Figure S2). To verify the reliability of equal‐weighted summation, a weighted predictive score base the standardized coefficients (1.99×hyperdensity+1.73×hypodensity) was compared with the Co‐ASPECTS, with a C‐statistic of 0.890 (95% CI, 0.863–0.917), nearly to the Co‐ASPECTS (0.889 [95% CI, 0.862–0.917]; DeLong test *P*=0.866) in the development cohort.

### Evaluation of Comprehensive ASPECTS

We screened variables potentially predictive of outcome in the development cohort and then determined their significance through multivariable logistic analysis. Variables with significant predictive value were incorporated into the basic model, which served as a reference for comparison with Co‐ASPECTS (Table S6). In receiver operating characteristic analyses, Co‐ASPECTS demonstrated superior discrimination compared with the basic model (C‐statistic, 0.889 [95% CI, 0.862–0.917] versus 0.799 [95% CI, 0.762–0.836]; DeLong test *P*<0.001) in the development cohort. Also, Co‐ASPECTS, which integrated both the hyperdensity and hypodensity signs, showed greatly improved performance compared with either sign alone (both DeLong test *P*<0.001) (Figure 2). Internal validation using 1000 bootstrap resamples was also conducted, showing an optimism estimate of −0.0008, with an optimism‐adjusted C‐statistic of 0.8885 versus an apparent C‐statistic of 0.8893.

**Figure 2:**
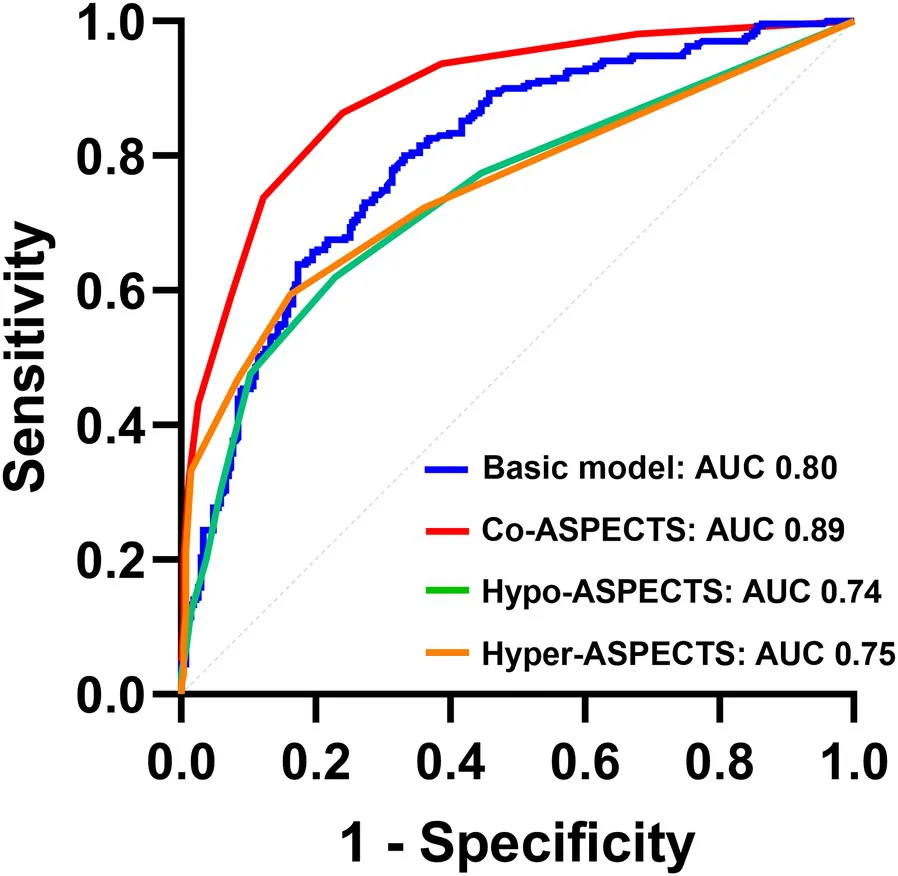
ROC analysis for functional outcome. Based on 90‐d functional outcome, 4 models were analyzed: basic model, hypo‐ASPECTS, hyper‐ASPECTS, and Co‐ASPECTS. AUC indicates area under the curve; Co‐ASPECTS, Comprehensive Alberta Stroke Program Early Computed Tomography Score; and ROC, receiver operating characteristic.

We further analyzed the net benefit of Co‐ASPECTS in differentiating between favorable and unfavorable functional outcome using net reclassification index and integrated discrimination improvement metrics. Results indicated that Co‐ASPECTS significantly enhanced the performance of both the simple and complex models (Table S7). The calibration curves of Co‐ASPECTS showed close alignment between predicted and actual outcome (Hosmer–Lemeshow test: χ^2^=5.23, *P*=0.515) (Figure 3), compared with ASPECTS (Figure S3). Decision curve analysis showed that the Co‐ASPECTS curve was above the horizontal, oblique lines and the basic model curve (Figure S4). Subgroup analyses demonstrated that Co‐ASPECTS maintained predictive performance and association strength across all subgroups (Figure S5).

**Figure 3:**
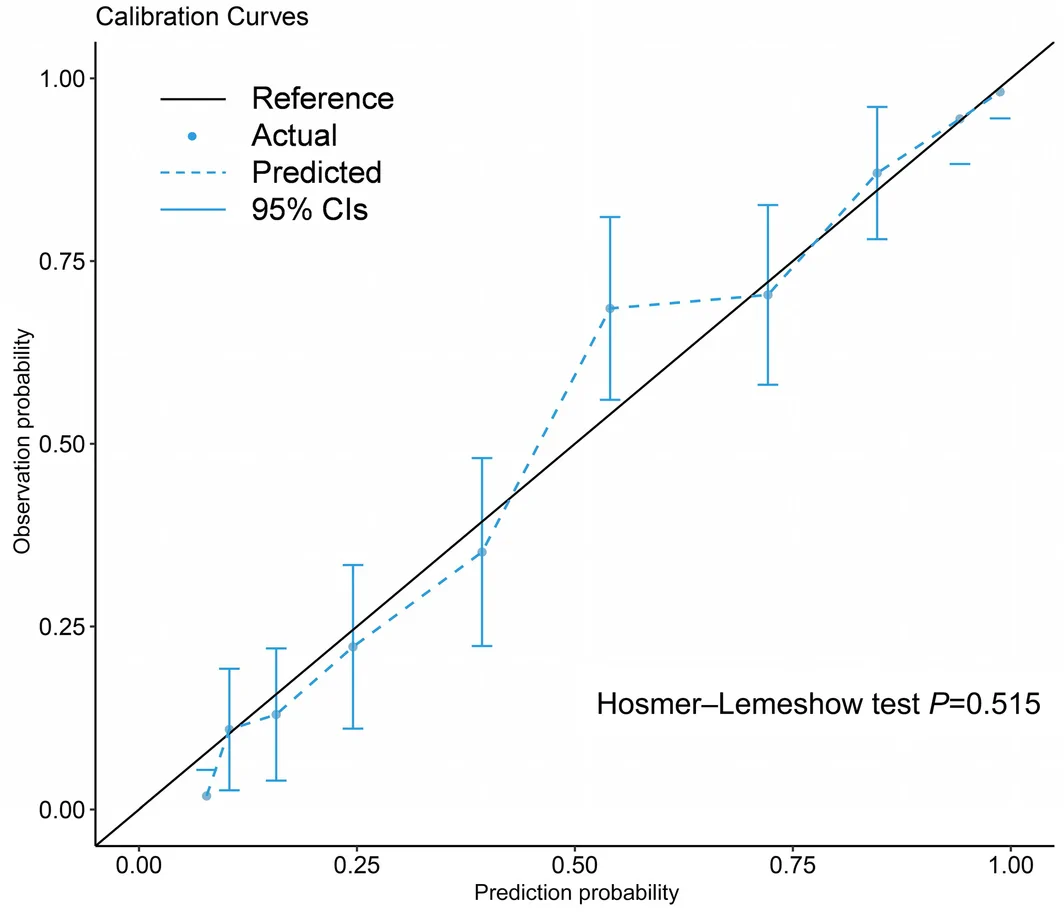
Calibration curve of Co‐ASPECTS for determining 90‐day functional outcome. The calibration curve compares the predicted probabilities with the actual outcomes. The diagonal line depicts ideal model accuracy. The Hosmer‐Lemeshow test sorted the predictions into deciles, assessing the discrepancies at each level (blue points) and their 95% CIs (blue lines). Co‐ASPECTS indicates Comprehensive Alberta Stroke Program Early Computed Tomography Score.

### External Validation of Comprehensive ASPECTS

To validate the accuracy and generalizability of Co‐ASPECTS, a national, multicenter, independent external validation was conducted. We first performed a sensitivity analysis, and the results showed that both signs had a significant impact on functional outcomes, and their weights remained approximately 1:1 (Table S8). Among the 473 patients from the external validation cohort, Co‐ASPECTS maintained the performance (C‐statistic, 0.847 [95% CI, 0.812–0.882]; Hosmer–Lemeshow test: χ^2^=9.50, *P*=0.218) (Figure S6A). To investigate whether the difference in baseline NIHSS score between the development cohort and the validation cohort affects the performance of Co‐ASPECTS, we selected patients with baseline NIHSS score ≥6 from the development cohort for subgroup analysis (C‐statistic, 0.894 [95% CI, 0.864–0.923], all DeLong test *P*<0.001; Hosmer–Lemeshow test: χ^2^=4.06, *P*=0.773) (Figure S6B). The distribution of Co‐ASPECTS scores and the percentage of poor functional outcome at each score showed no statistical difference across the development and validation cohorts (χ^2^=22, *P*=0.284 for score distribution; χ^2^=16, *P*=0.524 for poor outcome percentage) (Figure 4).

**Figure 4:**
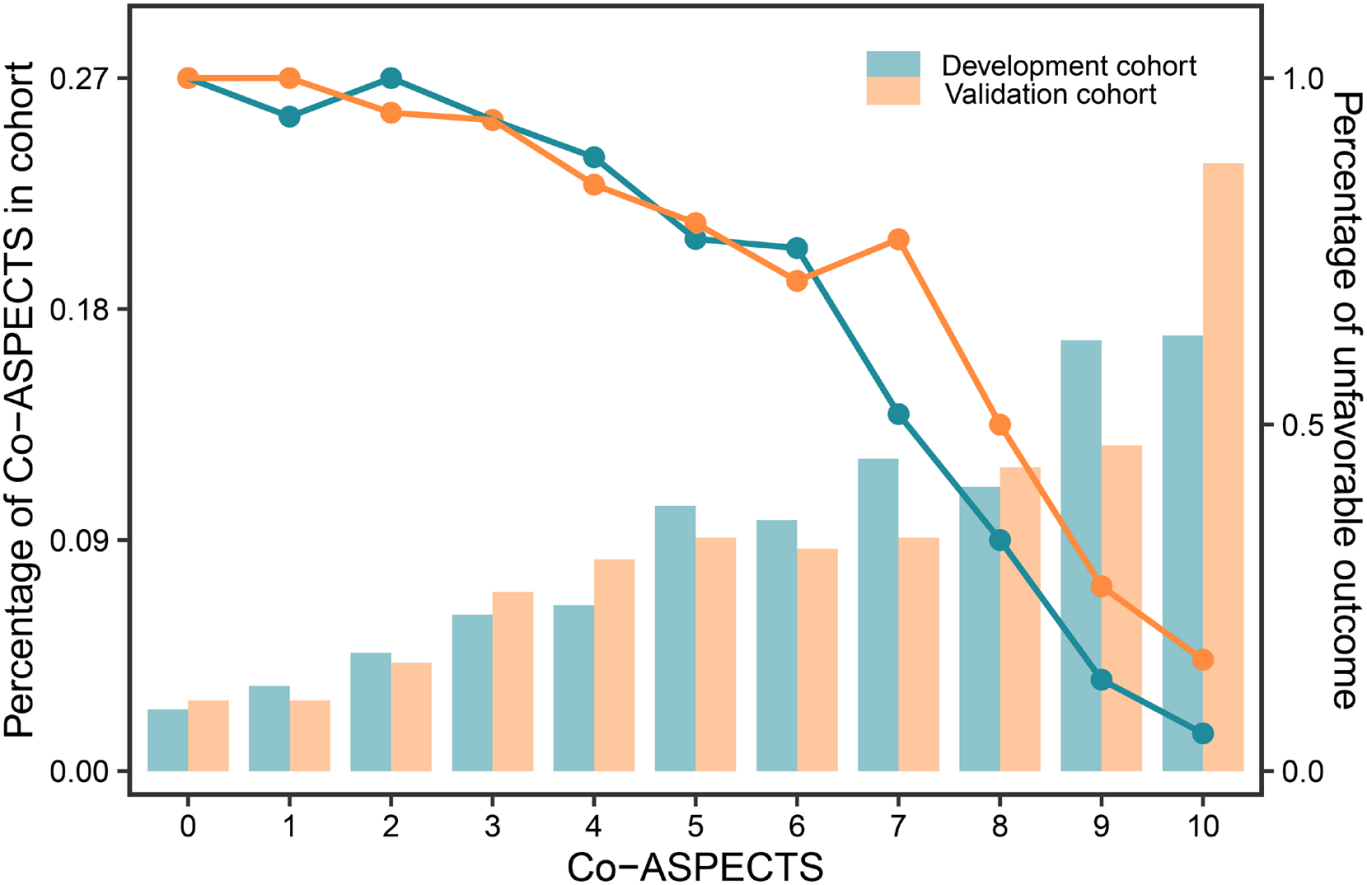
Distribution of each Co‐ASPECTS score and percentage of unfavorable functional outcome for each score in the development and validation cohorts. The horizontal axis represents the Co‐ASPECTS score, with higher scores indicating milder visible brain injury on immediate post‐EVT noncontrast CT. The barplots showed the proportion of patients in each cohort at each Co‐ASPECTS score, with the corresponding scale shown on the left vertical axis. The lineplots show the actual proportion of patients with unfavorable functional outcome at 90 d within each Co‐ASPECTS score, with the corresponding scale shown on the right vertical axis. Co‐ASPECTS indicates Comprehensive Alberta Stroke Program Early Computed Tomography Score; CT, computed tomography; and EVT, endovascular therapy.

In the overall population of 1015 patients, Co‐ASPECTS was compared head to head with potential internal optimizations and other previously reported models. Receiver operating characteristic analysis showed that Co‐ASPECTS achieved a C‐statistic of 0.866 (95% CI, 0.844–0.888), whereas the regression model was 0.860 (95% CI, [0.837–0.883]), and the 0 to 20 scale was 0.860 (95% CI, 0.838–0.883) (Figure S6C). Two universal models, PRE and THRIVE, were used for head‐to‐head comparison with Co‐ASPECTS in the overall population of 1015 patients. Co‐ASPECTS showed a higher C‐statistic (0.866 [95% CI, 0.844–0.888]) than PRE (0.754 [95% CI, 0.726–0.784]) and THRIVE (0.702 [95% CI, 0.671–0.734]; both *P*<0.001, DeLong test) (Figure S6D). The reclassification metrics of net reclassification index and integrated discrimination improvement also further favored Co‐ASPECTS (Table S7). The Brier score of Co‐ASPECTS was 0.128 in development cohort, 0.133 in the NIHSS score ≥6 subgroup, and 0.156 in validation cohort. For comparison, the basic model, PRE and THRIVE scores were 0.183, 0.201, and 0.217, respectively.

We also exploratorily identified the optimal cutoff value for Co‐ASPECTS in the 2 cohorts based on the Youden's index, which was 7.5 in both cohorts. Restricted cubic spline was employed to try to cautiously and exploratorily demonstrate the association between Co‐ASPECTS and functional outcome in the whole population (P for nonlinear=0.047), with the cutoff value between 7 and 8 (Figure S7). Based on this cutoff, both cohorts exhibited their specificity, sensitivity, positive predictive value, negative predictive value, and positive and negative likelihood ratios (Table 2). Additionally, we conducted a detailed risk analysis based on Co‐ASPECTS for predicting unfavorable outcome. The probability of poor outcome increased progressively as the Co‐ASPECTS decreases from 10 to 0: ∼9% for a score of 10, 48% for a score of 7, and >90% for a score <4. Notably, patients with hemorrhagic transformation had an elevated risk of poor outcome, and functional independence was likely unattainable when Co‐ASPECTS was <5, regardless of whether there was a hemorrhagic transformation or not (Figure 5).

**Table 2:** Predictive Performance of Co‐ASPECTS for Functional Outcome

	Development cohort N = 542	Validation cohort N = 473
C‐statistics (95% CI)	0.889 (0.862–0.917)	0.847 (0.812–0.882)
Cutoff value	7.5	7.5
Dichotomized test characteristics (95% Cl)		
Sensitivity	0.760 (0.709–0.811)	0.806 (0.752–0.860)
Specificity	0.863 (0.823–0.904)	0.768 (0.717–0.818)
Positive predictive value	0.848 (0.803–0.893)	0.728 (0.670–0.786)
Negative predictive value	0.783 (0.736–0.829)	0.837 (0.790–0.883)
Positive likelihood ratio	5.568 (4.097–7.567)	3.470 (2.762–4.360)
Negative likelihood ratio	0.278 (0.224–0.345)	0.253 (0.190–0.337)

Co‐ASPECTS indicates Comprehensive Alberta Stroke Program Early Computed Tomography Score.

**Figure 5:**
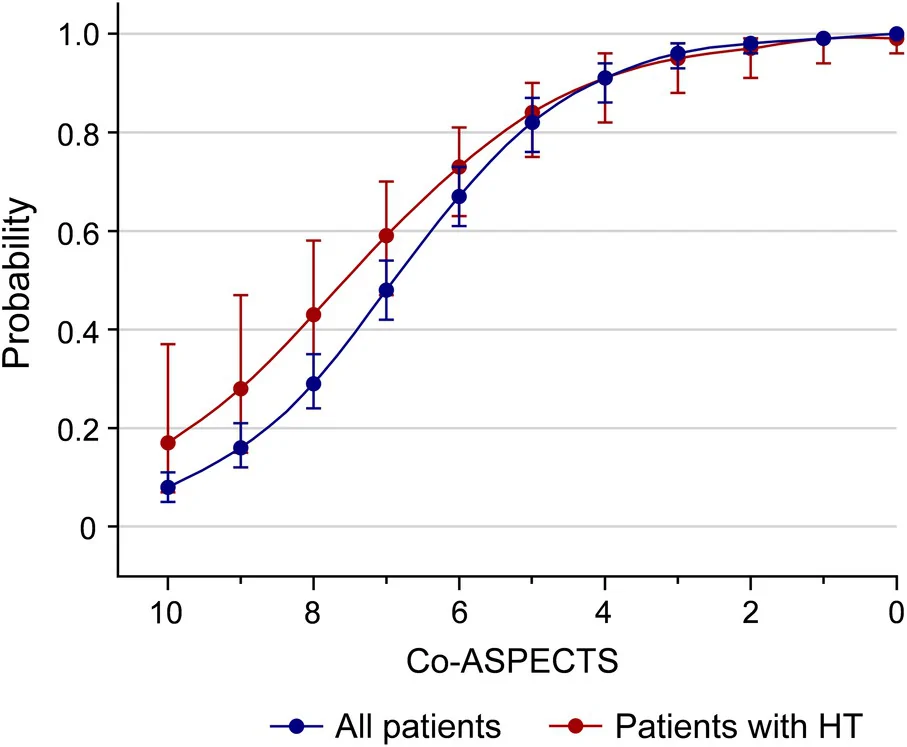
Logistic regression analysis to predict 90‐day unfavorable functional outcome. Probability for unfavorable functional outcome was according to Co‐ASPECTS in all patients and patients with HT. Co‐ASPECTS indicates Comprehensive Alberta Stroke Program Early Computed Tomography Score; and HT, hemorrhagic transformation.

### Interpretation of Comprehensive ASPECTS

The Co‐ASPECTS was derived from post‐EVT hypodensity and hyperdensity signs. To explore its potential mechanisms, we separately investigated the associations of post‐EVT hypodensity and hyperdensity with functional outcome, ultimately validating these findings through the Co‐ASPECTS. For hypodensity, the accuracy of immediate post‐EVT scans in predicting the final infarct size was significantly higher than that of baseline scans across all ASPECTS regions, which was 86.73%, suggesting a more precise reflection of primary infarction‐induced injury (Table S9). A performance comparison was also conducted between models that included only baseline hypodensity signs and that included both the baseline and post‐EVT. Metrics including area under the curve, Aikake information criterion, Bayesian information criterion, and R^2^ validated the increment after adding the post‐EVT (Table S10). We also calculated the discrepancy between post‐EVT and baseline hypodensity signs, which indicated a significant association with unfavorable outcome (adjusted OR, 1.66 [95% CI, 1.48–1.86], *P*<0.001) (Table S11). For hyperdensity, this sign was statistically significant associated with both intracranial hemorrhage and symptomatic intracranial hemorrhage, confirming its role as a predictor (Table S12).

Building on these findings, we further examined the relationship between Co‐ASPECTS and final infarction, symptomatic intracranial hemorrhage, and functional outcome, which revealed significant pairwise correlations (Figure 6). For the 93 patients with missing final infarction data, whose baseline and treatment characteristics did not reveal significant differences, a sensitivity analysis using multiple imputation was performed (Table S13). The results showed that the β value for Co‐ASPECTS and final infarction was −0.47 (95% CI, −0.50 to −0.43), and the OR for final infarction and poor outcomes was 1.10 (95% CI, 1.08 to 1.11).

**Figure 6:**
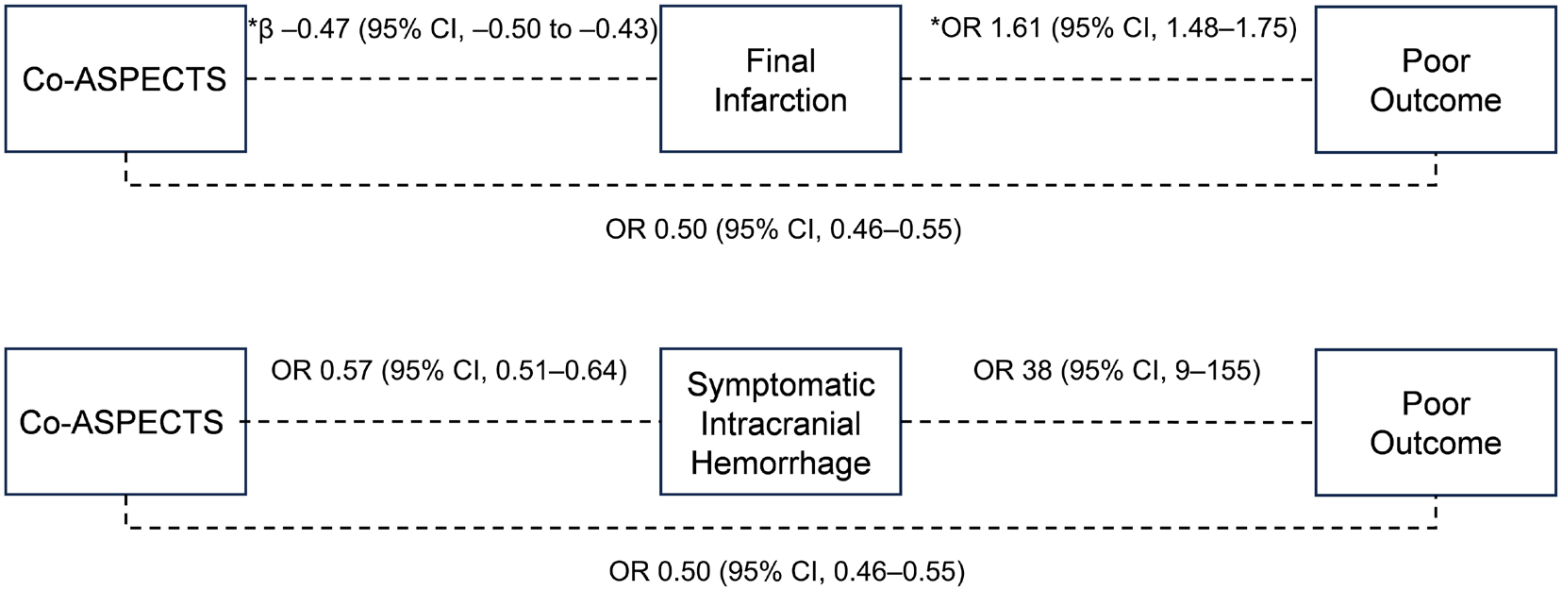
Relationships of Co‐ASPECTS, final infarction, symptomatic intracranial hemorrhage, and poor outcome. *The included population was 922 patients who actually underwent CT scans ∼24 (24±6) hours after EVT. The connections represent statistical correlations among the variables, not causal mediation pathways. Co‐ASPECTS indicates Comprehensive Alberta Stroke Program Early Computed Tomography Score; EVT, endovascular thrombectomy; and OR, odds ratio.

## DISCUSSION

In this study, we developed a novel predictive tool for patients with AIS undergoing EVT based on NCCT imaging performed immediately post EVT. The Co‐ASPECTS, which followed the scoring criteria of the ASPECTS system and incorporated both hyperdensity and hypodensity signs, demonstrated reliable discrimination and calibration and was validated in an independent national multicenter cohort.

For patients with AIS treated with EVT, the functional outcome not only depends on the severity of stroke onset, which can be measured by the size of the infarct volume, but also is closely related to the performance of recanalization therapy.^26^, ^27^ The hypodensity on Co‐ASPECTS captures infarct growth from baseline imaging to recanalization, serving as a dynamic extension and updated assessment of baseline ASPECTS that accurately reflects the true irreversible damage and final infarct burden.^28^ Moreover, post‐EVT hypodensity reflects either severe initial ischemia or periprocedural infarct growth. Both pathways end in irreversible necrosis, captured by Co‐ASPECTS as the key anatomical predictor of functional outcome.

Recanalization therapy in patients with AIS is another major factor affecting prognosis. BBB damage associated with EVT is a direct cause of contrast extravasation or hemorrhage into the brain parenchyma, often seen as a hyperdensity sign on post‐EVT CT imaging, mainly caused by medical interventions, including surgical operation and reperfusion injury.^29^ Reperfusion, although necessary for restoring blood flow, can exacerbate oxidative stress, trigger inflammatory response, and promote apoptosis in hypoxic tissues, all of which are strongly linked to BBB damage.^30^, ^31^ In addition, hyperdensity signs post EVT are associated with an increased risk of symptomatic intracranial hemorrhage and malignant brain edema.^32^, ^33^

In our study, the association between prognosis and both signs was equally strong, leading us to integrate them proportionally into a single scoring system. This 0 to10 scoring system showed performance with no statistical difference but much more simplicity in clinical practice than the coefficient‐weighted and direct 0 to 20 sum score system. Although hypodensity sign primarily reflects the extent of early established ischemic tissue damage, hyperdensity sign captures damage to the BBB and potential hemorrhagic conditions. Integrating these 2 complementary aspects seems to provide a multidimensional and more holistic baseline evaluation of the ischemic pathophysiology. The metrics used in Co‐ASPECTS were derived from NCCT imaging immediately within 1 hour post EVT, captures the earliest postreperfusion tissue status, which minimizing time‐dependent heterogeneity enables, and enables real‐time clinical decisions without waiting 24 hours. Moreover, immediate post‐EVT NCCT is routinely performed to rule out procedural complications, so Co‐ASPECTS adds no cost or workflow delay, ensuring real‐world utility. Additionally, the use of NCCT enhanced the model's real‐world applicability, given the accessibility in routine clinical settings. The simplicity and practicality of ASPECTS‐based scoring further ensure the feasibility of applying Co‐ASPECTS in clinical practice. The inclusion of patients with baseline NIHSS score ≥6, who were more likely to benefit from EVT, maintained robust predictive performance in both the development and validation cohorts. Regarding the decline in predictive performance from 0.889 in the development cohort to 0.847 in the validation cohort, we consider that the development cohort was drawn from 2 large academic hospitals, whereas the validation cohort was a multicenter study nationwide, resulting in greater heterogeneity among patients, medical teams, and other factors. Even so, the validation cohort still demonstrated acceptable performance.

The Co‐ASPECTS offers several features over other models. First, it could capture periprocedural infarct growth and reperfusion quality, providing an updated assessment at the time of recanalization. Second, it uses direct tissue biomarkers, including hypodensity for irreversible edema and hyperdensity for BBB disruption. Third, it integrates postprocedural hyperdensity as a predictor of poor outcome, adding a dimension of reperfusion injury absent in traditional models, although the value between symptomatic intracranial hemorrhage and poor outcome should be interpreted with caution because of the small number of events. Based on this, and as the decision curve analysis analysis showed, Co‐ASPECTS is expected to serve as an early warning signal in clinical practice. For low‐risk patients, it may guide resource optimization, including shorter intensive care unit stay and early rehabilitation. For high‐risk patients, it alerts practitioners to severe irreversible or secondary damage, prompting intensive monitoring, cautious blood pressure and antithrombotic management, early imaging, neurosurgical consultation, and family communication. Additionally, Co‐ASPECTS can stratify patients in clinical trials of neuroprotective or post‐EVT strategies. The cutoff of 7.5, which may lead to optimistic estimates, was considered an exploratory candidate for risk stratification rather than a definitive decision‐making threshold, and needs to be verified by other studies.

This study has several limitations. First, the model included only patients with acute anterior circulation large vessel occlusive stroke undergoing EVT, excluding posterior circulation or non‐EVT patients, though unconstrained recanalization status and baseline NIHSS score improved its target‐population generalizability. Second, only NCCT was adopted without CT perfusion/MRI confirmation, yet its clear imaging features and strong long‐term prognostic correlation guaranteed reliability. Third, the 2‐hospital development cohort may cause selection bias, whereas consistent efficacy in the national multicenter validation verified its stability. Fourth, single‐country data might restrict Co‐ASPECTS' international generalizability. However, the scoring system's reliance on standard NCCT and the globally familiar ASPECTS framework ensures its potential of feasibility and adoption, even in resource‐limited environments. Future international studies are essential to confirm its cross‐regional reliability and universal applicability.

## Conclusions

In conclusion, we developed a scoring system, Co‐ASPECTS, for predicting the functional outcome of patients with AIS who received EVT and confirmed its efficacy in the external validation cohort. Given its reliability and simplicity, Co‐ASPECTS offers a promising tool for assessing brain lesions and predicting long‐term functional outcome in patients with AIS following EVT.

## Sources of Funding

This work was supported by Jiangxi Province Natural Science Foundation (No. 20232ACB216008), and Clinical Research Project of Nanfang Hospital, Southern Medical University (No. 2025CR002 & 2022CR008).

## Disclosures

None.

## Supporting information

Tables S1–S13
Figures S1–S7
