# Supplementary material for: Assessing Brain Lesions and Predicting Functional Outcome in Patients With Anterior Circulation Stroke With Endovascular Therapy: Co‐ASPECTS
Source: J Am Heart Assoc. 2026 Aug 20;15(17):e051171. doi: 10.1161/JAHA.126.051171 (PMC13695824; doi:10.1161/JAHA.126.051171)
Supplement: Supplementary file 1 [file JAH3-15-e051171-s001.pdf]

Supplemental Material

Table S1. Patient Characteristics of Validation Cohort

Characteristics	Overall population (n=473)	90-Day mRS score ≤2 (n=206)	90-Day mRS score ≥3 (n=267)	P value
Age, median (IQR), y	66 (56-73)	61 (53-71)	67 (59-75)	<0.001*
Male, n (%)	325 (69)	142 (69)	183 (69)	0.927
Underlying diseases, n (%)				
Hypertension	293 (62)	130 (63)	163 (61)	0.648
Diabetes mellitus	142 (30)	65 (32)	75 (28)	0.413
Dyslipidemia	194 (41)	109 (53)	85 (32)	<0.001*
Prior stroke	71 (15)	30 (15)	41 (15)	0.811
Atrial fibrillation	128 (27)	48 (23)	80 (30)	0.106
Prestroke mRS score 0, n (%)	397 (84)	177 (86)	220 (82)	0.301
Received i.v. tPA, n (%)	130 (27)	55 (27)	75 (28)	0.737
Clinical parameters at admission				
SBP, median (IQR), mmHg	143 (132-156)	142 (131-158)	145 (133-155)	0.589
DBP, median (IQR), mmHg	81 (74-90)	82 (74-91)	80 (73-90)	0.525
Glucose, median (IQR), mmol/L	6.7 (5.8-8.8)	6.3 (5.5-7.9)	6.9 (6.1-9.1)	0.001*
NIHSS score, median (IQR)	13 (10-17)	11 (8-14)	15 (11-19)	<0.001*
ASPECTS, median (IQR)	8 (7-9)	9 (8-10)	7(6-9)	<0.001*
Occlusion location, n (%)				
ICA	90 (19)	46 (22)	44 (16)	0.108
MCA	320 (68)	142 (69)	178 (67)	0.602
ICA and MCA	63 (13)	18 (9)	45 (17)	0.010*
Onset-to-recanalization, median (IQR), min	545 (385-925)	563 (355-918)	544 (428-928)	0.473
Stroke subtype per TOAST, n (%)				
Large artery atherosclerosis	296 (63)	138 (67)	158 (59)	0.082
Cardioembolism	141 (30)	51 (25)	90 (34)	0.035*
Other	36 (8)	17 (8)	19 (7)	0.644
mTICI post-EVT				
0-2a	51 (11)	12 (6)	39 (15)	0.002*
2b-2c	123 (26)	47 (23)	76 (29)	0.917
3	299 (63)	147 (71)	152 (57)	0.001*

mRS, modified Rankin Scale; IQR, interquartile range; SBP, systolic blood pressure; DBP, diastolic blood pressure; NIHSS, National Institutes of Health Stroke Scale; ICA, internal cerebral artery; MCA, middle cerebral artery; TOAST, Trial of Org 10172 in Acute Stroke Treatment; ASPECTS, Alberta Stroke Program Early Computed Tomography Score; mTICI, modified Thrombolysis in Cerebral Infarction; EVT, endovascular therapy.

*Statistically significant

Table S2. Logistic Regression Analysis for the Association of Hyperdensity and Hypodensity Signs with Unfavorable Functional Outcome

	OR	95% CI	P value
**Model 1**			
Hyperdensity sign	2.36	2.00-2.79	<0.001*
Hypodensity sign	2.12	1.82-2.46	<0.001*
**Model 2**			
Hyperdensity sign	2.35	1.96-2.81	<0.001*
Hypodensity sign	2.23	1.89-2.63	<0.001*
**Model 3**			
Hyperdensity sign	2.53	2.07-3.09	<0.001*
Hypodensity sign	2.33	1.93-2.81	<0.001*

Model 1: Unadjusted.
Model 2: Adjusted for sex, time of onset-to-recanalization, baseline NIHSS, and mTICI post-EVT.
Model 3: Adjusted for sex, dyslipidemia, prestroke mRS score, time of onset-to-recanalization, baseline NIHSS, baseline ASPECTS, and mTICI post-EVT.
OR, odds ratio; CI, confidence interval; NIHSS, National Institutes of Health Stroke Scale; mRS, modified Rankin Scale; ASPECTS, Alberta Stroke Program Early Computed Tomography Score; mTICI, modified Thrombolysis in Cerebral Infarction.
*Statistically significant

Table S3. Subgroup Analysis of Logistic Regression for Unfavorable Outcomes Associated with Hyperdensity Signs With or Without Intracranial Hemorrhage

	OR	95% CI	P value
**Model 1**			
With ICH	1.95	1.48-2.56	<0.001*
Without ICH	1.83	1.47-2.28	<0.001*
**Model 2**			
With ICH	1.91	1.42-2.56	<0.001*
Without ICH	1.71	1.35-2.17	<0.001*
**Model 3**			
With ICH	1.88	1.32-2.66	<0.001*
Without ICH	1.95	1.47-2.59	<0.001*

Model 1: Unadjusted.
Model 2: Adjusted for sex, time of onset-to-recanalization, baseline NIHSS, and mTICI post-EVT.
Model 3: Adjusted for sex, dyslipidemia, prestroke mRS score, time of onset-to-recanalization, baseline NIHSS, baseline ASPECTS, and mTICI post-EVT.
ICH, intracranial hemorrhage; OR, odds ratio; CI, confidence interval; NIHSS, National Institutes of Health Stroke Scale; mRS, modified Rankin Scale; ASPECTS, Alberta Stroke Program Early Computed Tomography Score; mTICI, modified Thrombolysis in Cerebral Infarction.
Patients whose immediate hyperdensity visibly resolved or faded on the 24-hour scan were classified as contrast extravasation, whereas those with persistent or expanding hyperdensity were classified as having intracranial hemorrhage.
*Statistically significant

Table S4. Subgroup Analysis of patients without hyperdensity signs and hyperdensity signs Without Intracranial Hemorrhage

	Without hyperdensity signs	Hyperdensity signs without ICH	P value
Final infarction (OR, 95%CI)	*Ref.*	2.54 (1.88-3.43)	<0.001*
Unfavorable outcome (median, IQR)	1 (0-2)	1 (0-2)	0.211

ICH, intracranial hemorrhage; OR, odds ratio; CI, confidence interval; IQR, interquartile range.

*Statistically significant

Table S5. Additive Interaction of Hyperdensity and Hypodensity Signs on Functional Outcome

	Estimate	Lower 95% CI	Upper 95% CI
RERI	1.59	0.85	2.33
AP	0.33	0.25	0.41
SI	1.70	1.48	1.97

In the examination of additive interaction, the significance of RERI and AP indicated the validity of additive interaction. The SI was significantly greater than 1, indicating that the interaction between hyperdensity and hypodensity signs was positively synergistic.

CI, confidence interval; RERI, relative excess risk of interaction; AP, attributable proportion of interaction; SI, synergy index.

Table S6. Multivariable Logistic Regression Analysis Associated with Poor Functional Outcome (90-day mRS Score >2)

Parameters	Univariate analysis		Multivariate analysis	
	Odds ratio (95% CI)	P value	Odds ratio (95% CI)	P value
Male	1.54 (1.07-2.21)	0.021*	1.73 (1.11-2.69)	0.015*
Dyslipidemia	1.83 (1.25-2.67)	0.002*	1.79 (1.14-2.80)	0.011*
Prestroke mRS score	0.55 (0.34-0.87)	0.011*	0.47 (0.27-0.82)	0.008*
SBP	1.01 (1.00-1.01)	0.136		
Baseline NIHSS	1.13 (1.10-1.17)	<0.001*	1.15 (1.11-1.19)	<0.001*
Baseline ASPECTS	0.68 (0.61-0.77)	<0.001*	0.69 (0.60-0.79)	<0.001*
ICA and MCA occlusion	2.01 (1.20-3.38)	0.008*		
Onset-to-recanalization	1.00 (1.00-1.00)	0.010*	1.00 (1.00-1.00)	0.002*
Large artery atherosclerosis	0.73 (0.52-1.04)	0.077		
Cardioembolism	1.43 (0.97-2.12)	0.074		
mTICI post-EVT				
0-2a	*Ref.*	-	*Ref.*	-
2b-2c	0.58 (0.27-1.22)	0.151	0.35 (0.15-0.85)	0.021*
3	0.35 (0.17-0.71)	0.003*	0.25 (0.11-0.56)	0.001*

mRS, modified Rankin Scale; CI, confidence interval; SBP, systolic blood pressure; NIHSS, National Institutes of Health Stroke Scale; ASPECTS, Alberta Stroke Program Early Computed Tomography Score; ICA, internal cerebral artery; MCA, middle cerebral artery; mTICI, modified Thrombolysis in Cerebral Infarction; EVT, endovascular therapy.

*Statistically significant

Table S7. Predict Value of Co-ASPECTS for 90-Day Functional Outcome

	NRI		IDI	
	Estimate (95% CI)	P value	Estimate (95% CI)	P value
Model A+Co-ASPECTS	0.51 (0.41-0.61)	<0.001*	0.46 (0.42-0.50)	<0.001*
Model B+Co-ASPECTS	0.45 (0.36-0.55)	<0.001*	0.44 (0.40-0.48)	<0.001*
Model C+Co-ASPECTS	0.26 (0.18-0.34)	<0.001*	0.30 (0.27-0.34)	<0.001*
PRE+Co-ASPECTS	0.23 (0.17-0.30)	<0.001*	0.24 (0.21-0.26)	<0.001*
THRIVE+Co-ASPECTS	0.35 (0.28-0.41)	<0.001*	0.33 (0.30-0.36)	<0.001*

Model A: sex, age.
Model B: sex, dyslipidemia, prestroke mRS, mTICI post-EVT, time of onset-to-recanalization.
Model C: sex, dyslipidemia, prestroke mRS, mTICI post-EVT, time of onset-to-recanalization, baseline NIHSS, baseline ASPECTS.
CI, confidence interval; mRS, modified Rankin Scale; mTICI, modified Thrombolysis in Cerebral Infarction; EVT, endovascular therapy; NIHSS, National Institutes of Health Stroke Scale; ASPECTS, Alberta Stroke Program Early Computed Tomography Score.
*Statistically significant

Table S8. Subgroup Analysis of Hyperdensity and Hypodensity Sign for Poor Functional Outcome (90-day mRS Score >2)

Subgroups	Hyperdensity sign		Hypodensity Sign	
	Odds ratio	95% CI	Odds ratio	95% CI
Total	2.09	1.87, 2.34	1.91	1.73, 2.11
Sex				
Male	2.13	1.85, 2.46	1.95	1.73, 2.19
Female	2.05	1.72, 2.46	1.83	1.55, 2.17
Age				
<65	1.95	1.67, 2.27	1.87	1.64, 2.14
≥65	2.26	1.91, 2.67	1.96	1.69, 2.27
Baseline NIHSS				
<5	1.42	1.05, 1.93	1.83	1.31, 2.57
5-15	2.14	1.86, 2.47	1.91	1.68, 2.17
>15	2.18	1.71, 2.78	1.82	1.50, 2.21
Received i.v. tPA				
Yes	1.97	1.58, 2.47	1.77	1.47, 2.12
No	2.14	1.88, 2.44	1.97	1.75, 2.21
Cohort				
Development cohort	2.36	2.00, 2.79	2.12	1.82, 2.46
Validation cohort	1.93	1.65, 2.25	1.77	1.55, 2.03

mRS, modified Rankin Scale; CI, confidence interval; NIHSS, National Institutes of Health Stroke Scale.

Table S9. Comparison with the Accuracy of Final Infarction

	Overall accuracy, %	P value	Individual accuracy median (IQR), %	P value
Hypodensity sign at baseline*	82.42	<0.001	90 (80-90)	<0.001
Hypodensity sign post EVT*	86.73		90 (80-100)	
Hypodensity sign at baseline†	82.67	<0.001	90 (80-90)	<0.001
Hypodensity sign post EVT†	86.90		90 (80-100)	

Final infarction was assessed using the ASPECTS scoring system based on CT scans performed approximately 24 hours after EVT to compare the accuracy of hypodensity sign at baseline and hypodensity sign post EVT to the final infarction. Overall accuracy was assessed based on all ASPECTS regions, while individual accuracy was compared based on each patient's accuracy.
*The included population was 922 patients who actually underwent CT scans approximately 24 hours after EVT.
†The included population was all 1015 patients in this study. For the 93 patients for whom CT scans approximately 24 hours after EVT were unavailable, multiple imputation methods were employed for sensitivity analysis.

Table S10. Predictive Performance of Baseline and Combined Post-EVT Hypodensity Signs for Unfavorable Outcome

	R^2	AIC	BIC	AUC
Baseline hypodensity Signs	0.10	1272	1281	0.71
Combined post-EVT hypodensity signs	0.17	1167	1177	0.77

Table S11. Logistic Regression Analysis for Unfavorable Outcomes With the Increment Between Post-EVT Hypodensity Signs and Baseline Hypodensity Signs

	OR	95% CI	P value
**Model 1**	1.10	1.03-1.16	0.002*
**Model 2**	1.09	1.02-1.18	0.010*
**Model 3**	1.66	1.48-1.86	<0.001*

Model 1: Unadjusted.
Model 2: Adjusted for sex, time of onset-to-recanalization, baseline NIHSS, and mTICI post-EVT.
Model 3: Adjusted for sex, dyslipidemia, prestroke mRS score, time of onset-to-recanalization, baseline NIHSS, baseline ASPECTS, and mTICI post-EVT.
ICH, intracranial hemorrhage; OR, odds ratio; CI, confidence interval; NIHSS, National Institutes of Health Stroke Scale; mRS, modified Rankin Scale; ASPECTS, Alberta Stroke Program Early Computed Tomography Score; mTICI, modified Thrombolysis in Cerebral Infarction.
*Statistically significant

Table S12. Logistic Regression Model of ICH and sICH for Hyperdensity Sign post-EVT and Co-ASPECTS

	ICH			sICH		
	Odds ratio	95% CI	P value	Odds ratio	95% CI	P value
Univariable						
Hyperdensity sign	1.93	1.76-2.11	<0.001	2.08	1.83-2.35	<0.001
Co-ASPECTS	0.66	0.62-0.70	<0.001	0.57	0.51-0.64	<0.001
Multivariable						
Hyperdensity sign	1.84	1.68-2.02	<0.001	2.09	1.83-2.39	<0.001
Co-ASPECTS	0.68	0.63-0.73	<0.001	0.56	0.50-0.63	<0.001

In the multivariate logistic regression model, gender, age, intravenous thrombolysis, baseline NIHSS, baseline ASPECTS, and occluded vessels were adjusted.

ICH, intracranial hemorrhage; sICH, symptomatic intracranial hemorrhage; EVT, endovascular therapy; CI, confidence interval.

Table S13. Patient Characteristics With or Without 24-Hour Post-EVT CT Scan

Characteristics	with the data (n=922)	without the data (n=93)	P value
Age, median (IQR), y	65 (56-73)	65 (57-73)	0.831
Male, n (%)	635 (69)	61 (66)	0.515
Underlying diseases, n (%)			
Hypertension	556 (60)	58 (62)	0.698
Diabetes mellitus	259 (28)	28 (30)	0.681
Dyslipidemia	319 (35)	28 (30)	0.384
Prior stroke	139 (15)	16 (17)	0.587
Atrial fibrillation	201 (22)	25 (27)	0.262
Prestroke mRS score 0, n (%)	777 (84)	74 (80)	0.240
Received i.v. tPA, n (%)	230 (25)	23 (25)	0.964
Clinical parameters at admission			
SBP, median (IQR), mmHg	144 (131-158)	136 (127-155)	0.125
DBP, median (IQR), mmHg	82 (75-92)	81 (70-89)	0.102
Glucose, median (IQR), mmol/L	6.9 (5.8-8.8)	6.7 (5.7-8.1)	0.233
NIHSS score, median (IQR)	12 (8-16)	11 (8-16)	0.557
ASPECTS, median (IQR)	8 (7-9)	8 (7-9)	0.642
Occlusion location, n (%)			
ICA	214 (23)	24 (26)	0.573
MCA	588 (64)	55 (59)	0.377
ICA and MCA	120 (13)	14 (15)	0.580
Onset-to-recanalization, median (IQR), min	550 (377-928)	499 (369-818)	0.269
Stroke subtype per TOAST, n (%)			
Large artery atherosclerosis	577 (63)	55 (59)	0.514
Cardioembolism	247 (27)	28 (30)	0.493
Other	98 (11)	10 (11)	0.971
mTICI post-EVT			
0-2a	85 (9)	6 (6)	0.373
2b-2c	243 (26)	23 (25)	0.734
3	594 (64)	64 (69)	0.398
Co-ASPECTS, median (IQR)	7 (5-9)	7 (5-9)	0.910
90-Day mRS 0-2, n (%)	429 (47)	48 (52)	0.349

mRS, modified Rankin Scale; IQR, interquartile range; SBP, systolic blood pressure; DBP, diastolic blood pressure; NIHSS, National Institutes of Health Stroke Scale; ICA, internal cerebral artery; MCA, middle cerebral artery; TOAST, Trial of Org 10172 in Acute Stroke Treatment; ASPECTS, Alberta Stroke Program Early Computed Tomography Score; mTICI, modified Thrombolysis in Cerebral Infarction; EVT, endovascular therapy.

*Statistically significant

Figure S1. Patient inclusion flowchart.

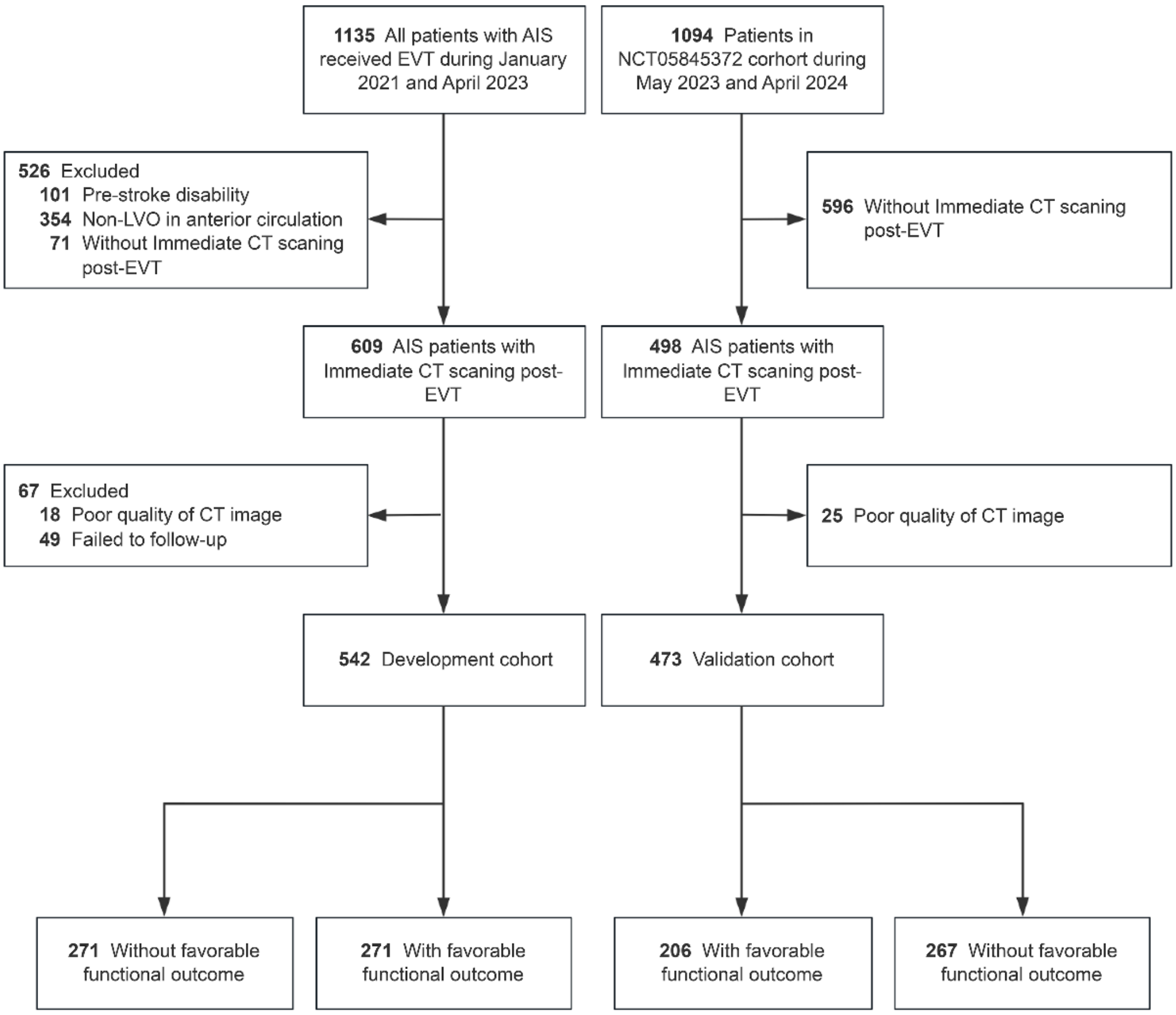

Figure S1. Patient inclusion flowchart.

Figure S2. Relationship between hyperdensity and hypodensity signs in the ASPECTS-regions in the total population.

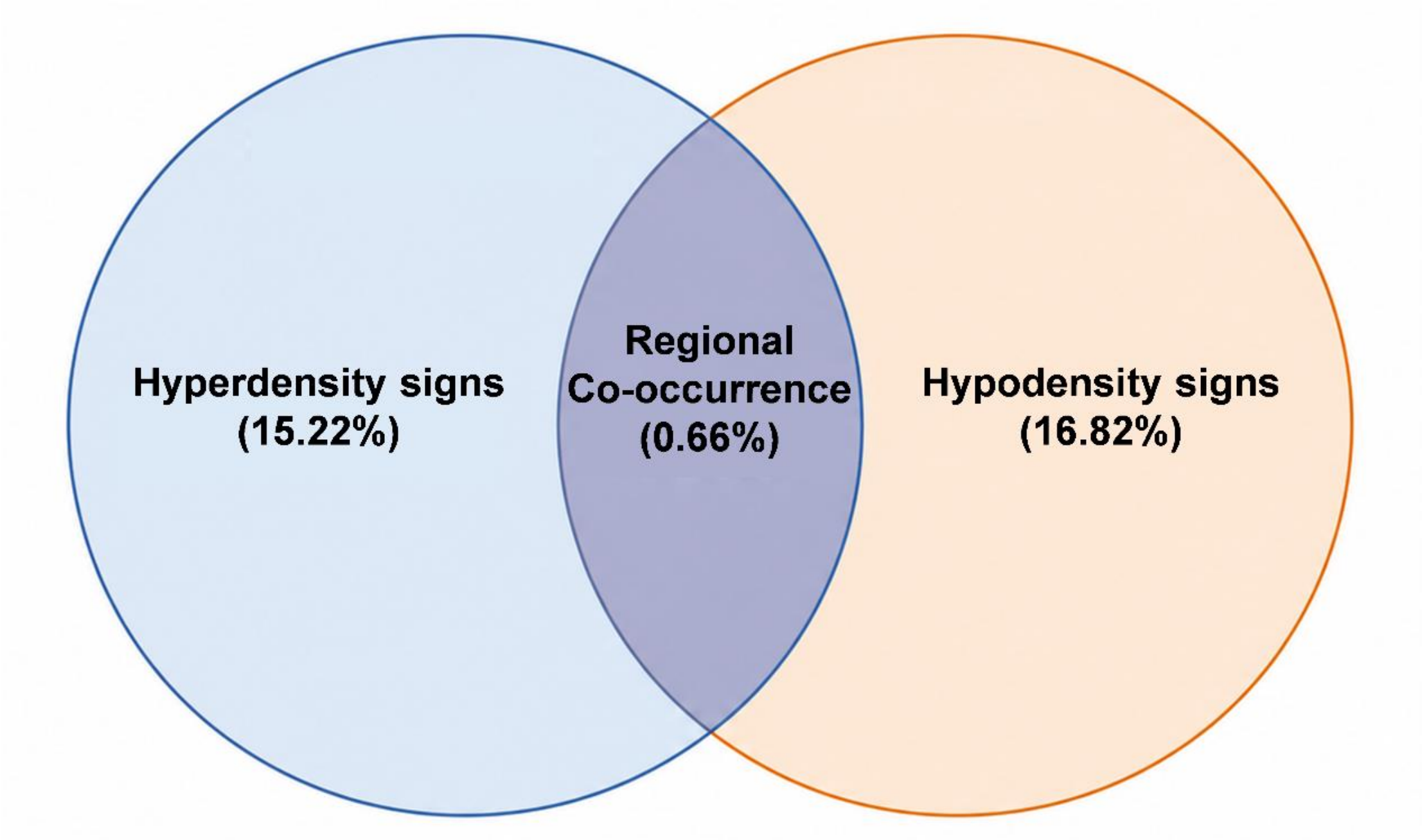

Figure S2. Relationship between hyperdensity and hypodensity signs in the ASPECTS-regions in the total population. According to the ASPECTS scoring system, the NCCT images of each patient after EVT were divided into 10 regions. It showed the proportion and relationship of hyperdensity and hypodensity signs in the total regions.

Figure S3. Calibration curve of ASPECTS for determining 90-day functional outcome.

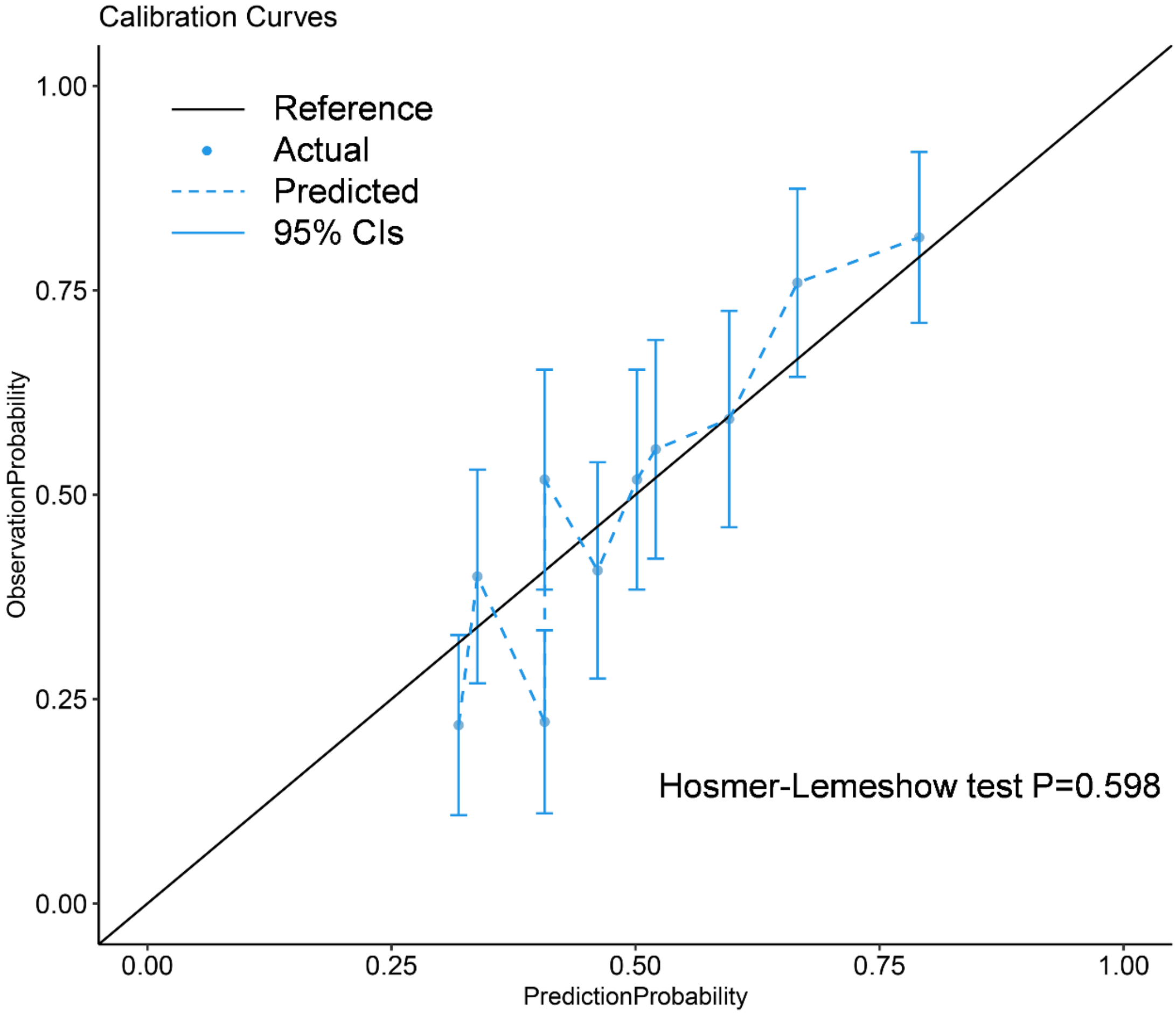

Figure S3. Calibration curve of ASPECTS for determining 90-day functional outcome. The calibration curve compares the predicted probabilities with the actual outcomes. The diagonal line depicted ideal model accuracy. The Hosmer-Lemeshow test sorted the predictions into deciles, assessing the discrepancies at each level (blue points) and their 95% confidence intervals (blue lines).

Figure S4. Decision curve analyses depicting the clinical benefit of predictive models.

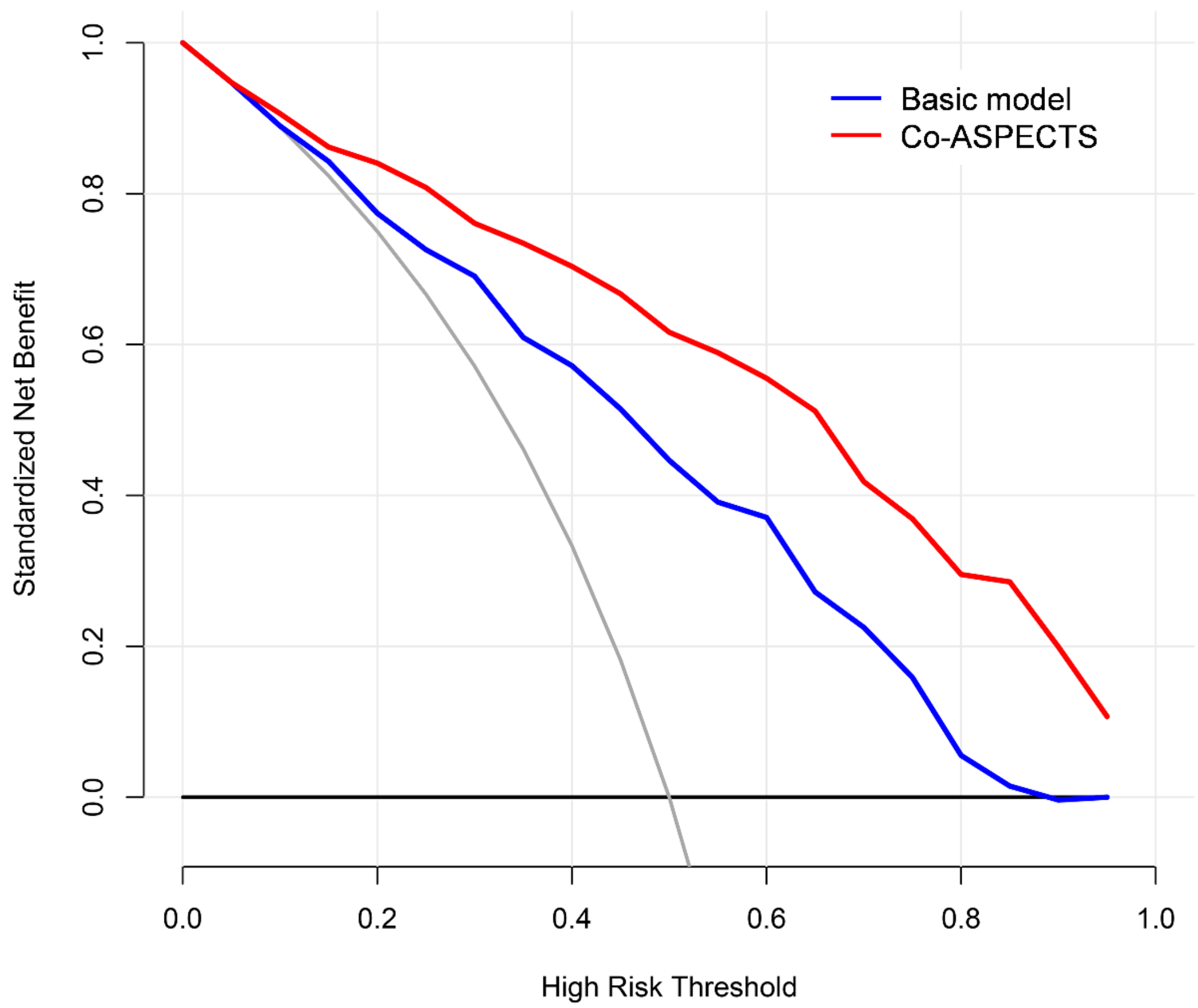

Figure S4. Decision curve analyses depicting the clinical benefit of predictive models. The Co-ASPECTS provided more accuracy than the basic model, treat all, and treat none, with a wide threshold probability range and a positive net return.

Figure S5. The effect of Co-ASPECTS on functional outcome in different subgroup.

Subgroup	Number	OR (95% CI)	P value	AUC
All Patients	542	0.45 (0.40 to 0.51)	<0.001	0.889
Sex				
Male	371	0.43 (0.36 to 0.50)	<0.001	0.893
Female	171	0.49 (0.40 to 0.60)	<0.001	0.886
Age				
<65 yr	265	0.49 (0.42 to 0.58)	<0.001	0.869
>65 yr	277	0.41 (0.33 to 0.50)	<0.001	0.908
Hemorrhagic transformation				
Yes	146	0.52 (0.40 to 0.68)	<0.001	0.835
No	396	0.45 (0.38 to 0.52)	<0.001	0.880
Occlusion location				
ICA	148	0.42 (0.32 to 0.55)	<0.001	0.908
MCA	323	0.45 (0.38 to 0.54)	<0.001	0.882
ICA+MCA	71	0.50 (0.37 to 0.69)	<0.001	0.858
Successful recanalization				
Yes	502	0.43 (0.38 to 0.50)	<0.001	0.892
No	40	0.61 (0.44 to 0.85)	0.004	0.827
Baseline NIHSS				
<5	84	0.62 (0.47 to 0.81)	<0.001	0.829
5−15	335	0.41 (0.34 to 0.49)	<0.001	0.891
>15	123	0.46 (0.34 to 0.61)	<0.001	0.900

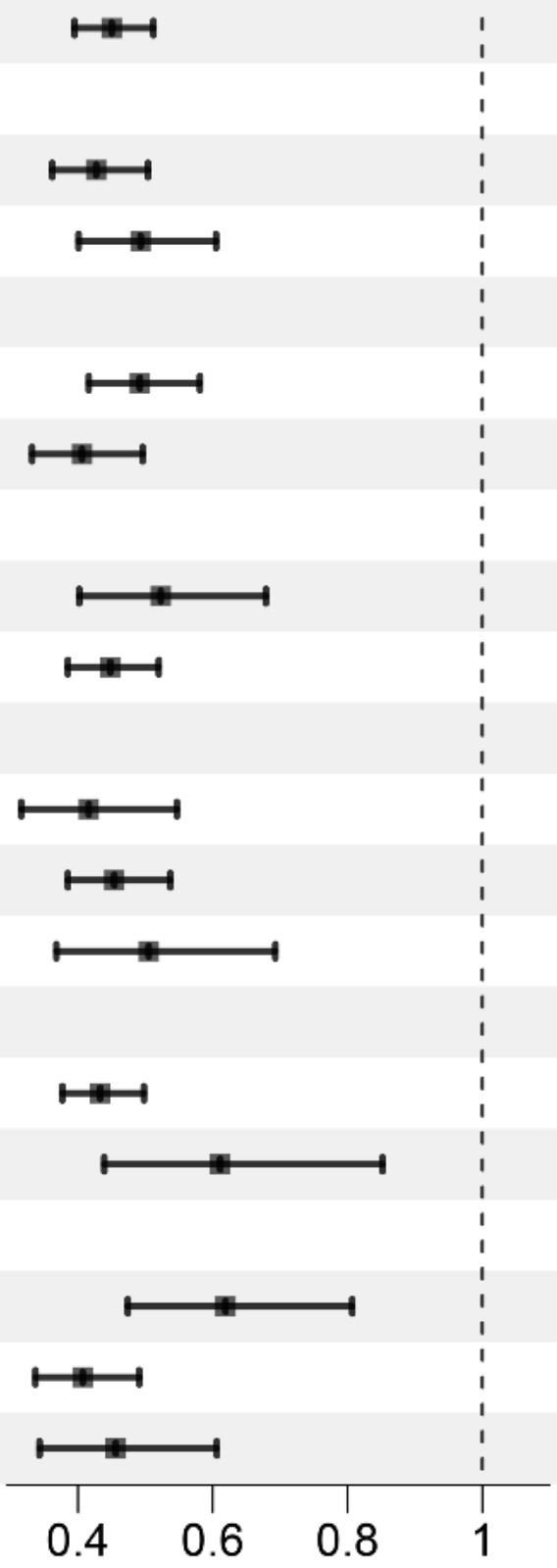

Figure S5. The effect of Co-ASPECTS on functional outcome in different subgroup. Forest plot presented the relative relation of OR in different subgroups for functional outcome. The black points in forest plot indicated the estimate OR, and the black lines indicated the 95% Cl.

Figure S6. ROC analysis for functional outcome with Co-ASPECTS.

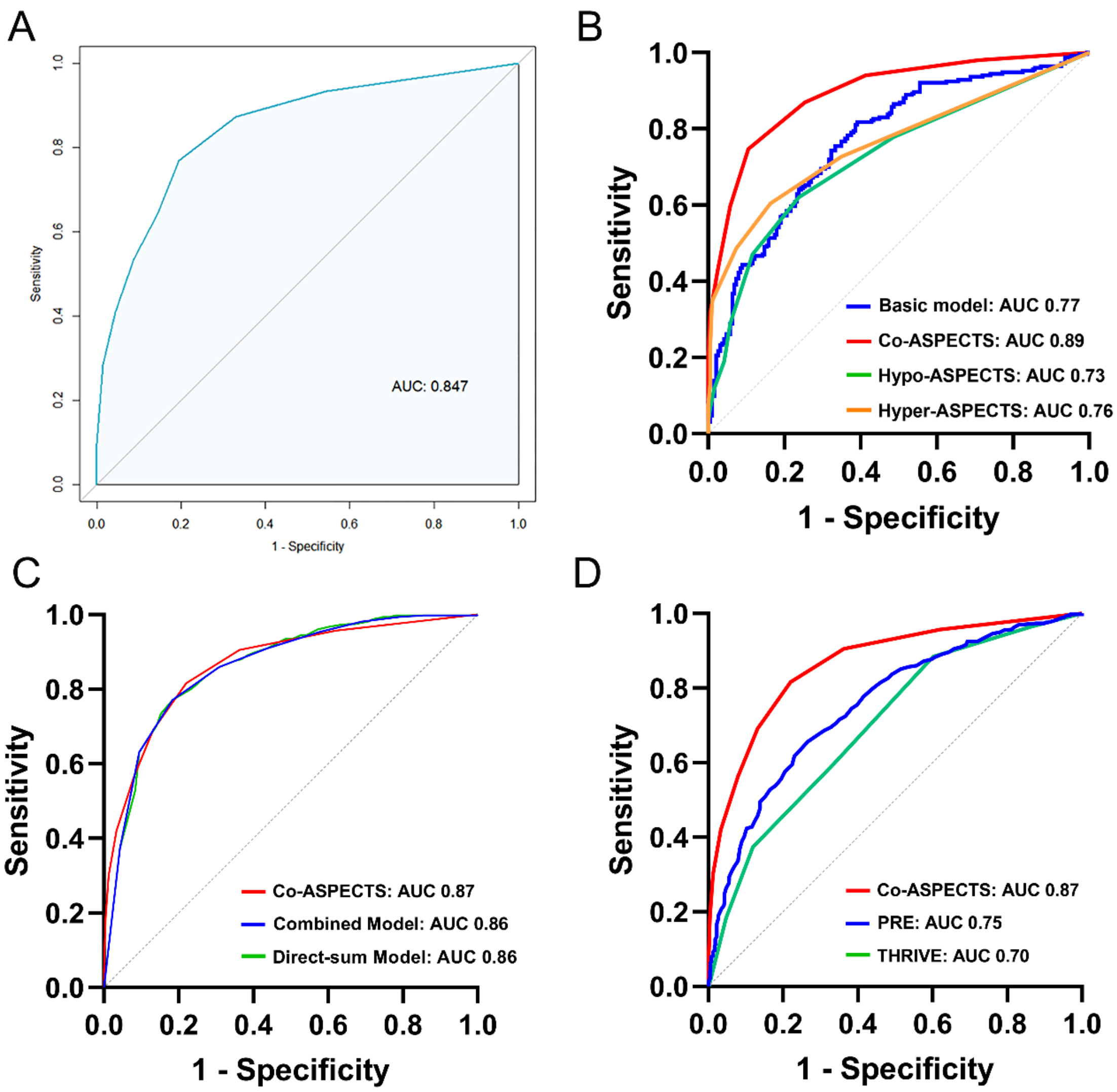

Figure S6. ROC analysis for functional outcome with Co-ASPECTS. A, ROC analysis for functional outcome with Co-ASPECTS in the validation cohort. B, ROC analysis for functional outcome in patients with baseline NlHSS ≥6 from the development cohort. Based on 90-day functional outcome, 4 models were analyzed: basic model, hypo-ASPECTS, hyper-ASPECTS, and Co-ASPECTS. C, ROC analysis for functional outcome in models with different calculation methods. The Co-ASPECTS score was calculated using a 0–10 scale, as described in the manuscript. The combined model integrated hyperdensity and hypodensity signs through regression analysis. The direct-sum model was a 0–20 scale derived by simply adding the counts of hyperdensity and hypodensity signs. D, ROC analysis for functional outcome among Co-ASPECTS, PRE Score, and Thrive Score.

Figure S7. Restricted cubic spline plots for the associations of Co-ASPECTS and 90-day functional outcome.

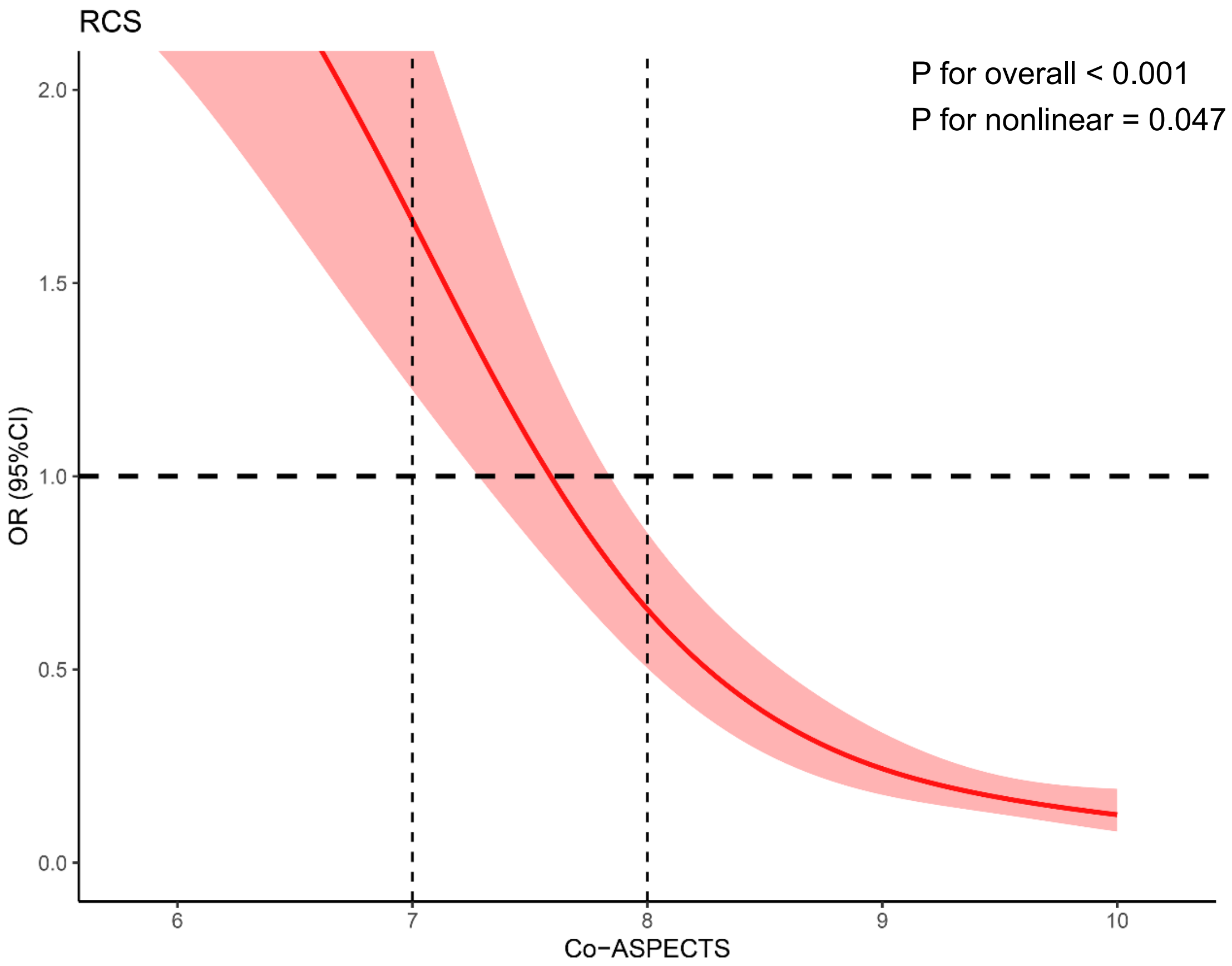

Figure S7. Restricted cubic spline plots for the associations of Co-ASPECTS and 90-day functional outcome. The red curve indicated the estimated value, and the light red shaded area indicated the 95% confidence interval.
